# Detection of Sugar in the Expectoration of Patients Affected with Diabetes

**Published:** 1847-04

**Authors:** 


MANCHESTER PATHOLOGICAL SOCIETY.

*Detection of Sugar in the Expectoration of Patients affected with Diabetes.*—Dr. Francis presented to the Society a specimen of sugar which he had obtained, a few days previously, from the expectoration of a man the subject of diabetes mellitus.

The patient, aged 25, for upwards of a year suffering the ordinary symptoms of this disease, and at present much wasted in flesh, had, during the last six months, shown signs of advancing pulmonary phthisis. The expectoration latterly had amounted to little less than

24 ounces daily, and, on the day which furnished the specimen submitted to examination, had even exceeded that quantity. It was composed of an abundant white, frothy, tenacious mucus, holding in suspension little rounded masses of opaque yellow material.

In order to the detection of sugar, the expectoration was, first of all, treated freely with strong alcohol, which coagulated much of the albuminous matters. Distilled water was then added, and, after agitation and digestion for a short time, the whole was thrown upon a filter, and a clear watery fluid readily passed through.

A small portion of this fluid reduced the protoxide of copper when tested after the manner recommended by Trommer, and another portion underwent fermentation over mercury.

The remainder was evaporated in a water-bath to dryness, the residue broken up into fragments, and digested for several hours in alcohol, which was then filtered. The alcoholic solution thus obtained was of a yellowish tint, clear, and decidedly sweet to the taste. On evaporation, it left the considerable quantity of sugar now produced to the Society, and which will be found partly crystalline, of a rich sienna brown colour, strong honey-like odour, and intensely sweet taste.

A fluid ounce of the expectoration, after dilution with water, yielded by fermentation a trifle more than 2½ cubic inches of carbonic acid, which would be equivalent to 2½ grains of sugar, or 50 grains to the imperial pint.

The urine passed at the time of the examination contained sugar; its specific gravity was 1032, and its average standard for some days has been about 1035. The quantity passed was much less than formerly.

Dr. Francis had detailed at length the account of the process he had used, because, so far as he knew, the presence of sugar in the expectoration of diabetes had not previously been sought; at any rate, he could find no allusion to the subject in the Sydenham Society's edition of Simon's Animal Chemistry, which, with the notes of its accomplished editor, may be assumed to have brought our knowledge in such matters up to the present time.

In addition to the above case, he had, within the last two days, had the opportunity of examining the expectoration of another man who was under treatment two years ago with diabetes, and who, in addition to this, is now far advanced in phthisis. Here the expectoration was more scanty, and consisted of purulent matter, rendered tenacious by an admixture of rust-coloured secretion from a little local pneumonia. In this case an ounce of sputa contained so much as about seven grains of sugar.

It might be found, he thought, when closer attention came to be given to the subject, that there were other organs than the kidneys habitually playing an active part in the removal of the sugar which was accumulating in the blood during the progress of diabetes. There were, at least, some grounds for believing such might be the case from the results just detailed, and, if so, the quantity of sugar escaping in the urine could not be viewed as an absolutely safe index to the quantity formed in the system, unless taken in conjunction with other means of its elimination.

The cases might further be looked upon as furnishing an argument, if further evidence upon the subject were necessary, that the kidneys play no part in the formation, but merely in the separation from the blood, of the sugar.

